# Molecular-Based Taxonomic Inferences of Some Spider Mite Species of the Genus *Oligonychus* Berlese (Acari, Prostigmata, Tetranychidae)

**DOI:** 10.3390/insects14020192

**Published:** 2023-02-15

**Authors:** Hafiz Muhammad Saqib Mushtaq, Amgad A. Saleh, Muhammad Kamran, Fahad Jaber Alatawi

**Affiliations:** 1 Acarology Research Laboratory, Department of Plant Protection, College of Food and Agriculture Sciences, King Saud University, P.O. Box 2460, Riyadh 11451, Saudi Arabia; 2 Plant Pathology Laboratory, Department of Plant Protection, College of Food and Agriculture Sciences, King Saud University, P.O. Box 2460, Riyadh 11451, Saudi Arabia

**Keywords:** *Oligonychus*, ITS2, mtCOI, phylogeny, spider mites, species complex, taxonomy

## Abstract

**Simple Summary:**

Spider mites belonging to the genus *Oligonychus* Berlese are serious pests of various fruits, ornamentals, agronomic crops, and trees. To effectively manage these pests, their accurate identification is crucial. Morphological-based identification of *Oligonychus* species is very challenging. The present study used molecular data to identify/confirm the species identity of some *Oligonychus* species, including various samples lacking male specimens. Moreover, phylogenetic analyses validated the morphological-based subdivision of the genus *Oligonychus*. The integrative taxonomic approaches are vital for accurately identifying closely related *Oligonychus* species.

**Abstract:**

DNA barcoding technology using short DNA sequences has emerged as an efficient and reliable tool for identifying, confirming, and resolving closely related taxa. This study used ITS2-rDNA and mtCOI DNA sequences to confirm the identity of eight *Oligonychus* species, representing 68 spider mite samples, collected mainly from Saudi Arabia (SA) and some from Mexico, Pakistan, USA, and Yemen. The intraspecific nucleotide divergences of the studied *Oligonychus* species ranged from 0% to 1.2% for ITS2 and 0% to 2.9% for COI. However, the interspecific nucleotide divergences were distinctly higher than the intraspecific ones and ranged from 3.7% to 51.1% for ITS2 and 3.2% to 18.1% for COI. Furthermore, molecular data correctly confirmed the species identity of 42 *Oligonychus* samples lacking males, including a previously claimed sample of *O. pratensis* from SA. High genetic variations were detected in two *Oligonychus* species: *O. afrasiaticus* (McGregor) (nine ITS2 and three COI haplotypes) and *O. tylus* Baker and Pritchard (four ITS2 and two COI haplotypes). In addition, ITS2- and COI-based phylogenetic trees confirmed the subdivision of the genus *Oligonychus*. In conclusion, integrative taxonomic approaches are vital to resolve the closely related *Oligonychus* species, identify the samples lacking male specimens, and assess phylogenetic relationships within and among species.

## 1. Introduction

In the genus *Oligonychus* Berlese (Acari, Prostigmata, Tetranychidae), morphological-based differentiation among species always depends on the characterization of the key trait of male aedeagus [1,2,3]; however, in the subdivision of *Oligonychus* into subgenera and groups/subgroups, the characterization of both male and female is compulsory [3]. Indeed, exact species identification in *Oligonychus* is usually a challenge because of the minute differences in aedeagus; limited diagnostic traits in females; and the presence of various species complexes, e.g., *pratensis* complex and *punicae* complex [1,2,3,4,5,6,7]. Moreover, the key diagnostic trait of the aedeagus becomes unreliable if males are not mounted accurately in their lateral positions, or are either described briefly without illustrations or with insufficient morphological details [8,9]. Furthermore, the identity of some *Oligonychus* species remains questionable when these species were described based solely on females, and males were absent in the original and subsequent descriptions [10,11]. Therefore, these questionable *Oligonychus* species are considered *species inquirendae* [3].

Although morphological-based species identification is the most popular method used by biologists, especially taxonomists, it needs to be fortified by other methods, e.g., DNA-based ones, to be able to delimit species boundaries of closely related species. DNA-based methods include DNA sequences, e.g., nuclear and mitochondrial genes. The mitochondrial cytochrome *c* oxidase subunit I (COI) gene has been used extensively in identifying organisms, including mites [7,12,13,14,15]. The internal transcribed spacers (ITS1 and ITS2) regions of nuclear ribosomal DNA have also been applied accurately for the identification or confirmation of closely related species in the family Tetranychidae Donnadieu [7,13,14,16,17]. The combined use of morphological- and molecular-based methods for species delineation would greatly help resolve problems of synonymy and the misidentification of closely related tetranychid species [18,19,20]. Recently, an integrative taxonomic study has successfully resolved the long-standing issue of the *punicae* species complex in the genus *Oligonychus* [7].

Globally, 212 *Oligonychus* species have been reported so far [3,21,22]. In Saudi Arabia (SA), previous morphotaxonomic studies have revealed the presence of seven *Oligonychus* species, viz. *O. afrasiaticus* (McGregor), *O. coniferarum* (McGregor), *O. dactyloni* (Smiley and Baker), *O. punicae* (Hirst), *O. pratensis* (Banks), *O. tylus* (Baker and Pritchard), and *O. washingtoniae* (Mushtaq, Kamran, and Alatawi) [21,23]. However, the presence of the banks grass mite *O. pratensis* in SA could be doubtful due to the involvement of the *pratensis* species complex [1,21].

The present study aimed to apply the DNA-based methods to (1) confirm the morphologically identified *Oligonychus* species previously reported in SA, (2) identify mite samples that lack males, (3) investigate the intra- and inter-specific genetic variations of some widely distributed *Oligonychus* species, and (4) validate the subdivision of the genus *Oligonychus* into two subgenera.

## 2. Materials and Methods

### 2.1. Collection, Preservation, and Processing of Spider Mite Samples

**Figure 1:**
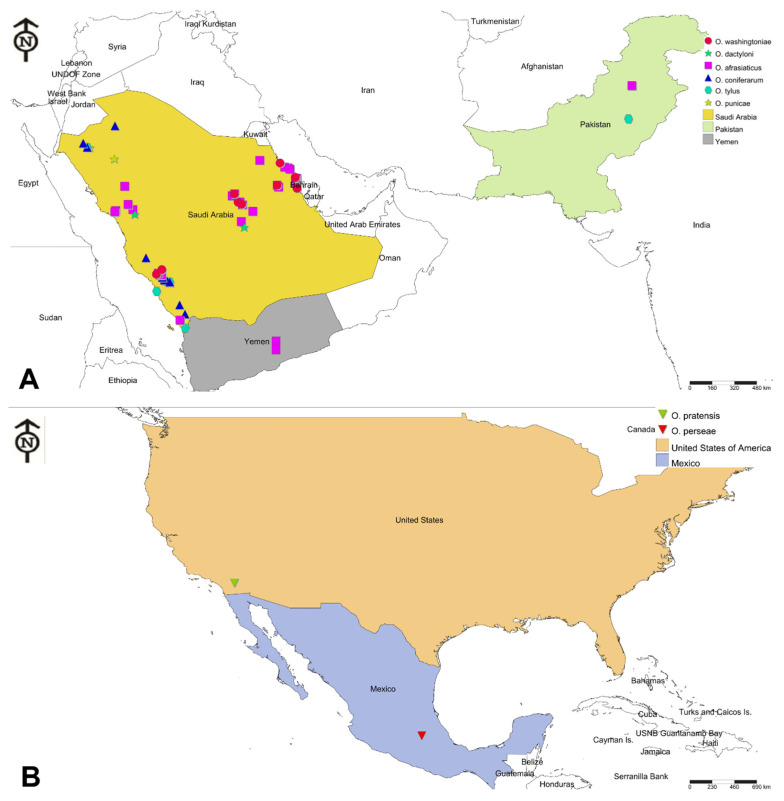
Geographical distribution of the (**A**) 66 spider mite samples of *Oligonychus afrasiaticus*, *O. coniferarum*, *O. dactyloni*, *O. punicae*, *O. tylus*, and *O. washingtoniae* along with the (**B**) two samples of *O. pratensis* and *O. perseae*, collected from various hosts and localities in Mexico, Pakistan, Saudi Arabia, United States, and Yemen in the present study.

**Figure 2:**
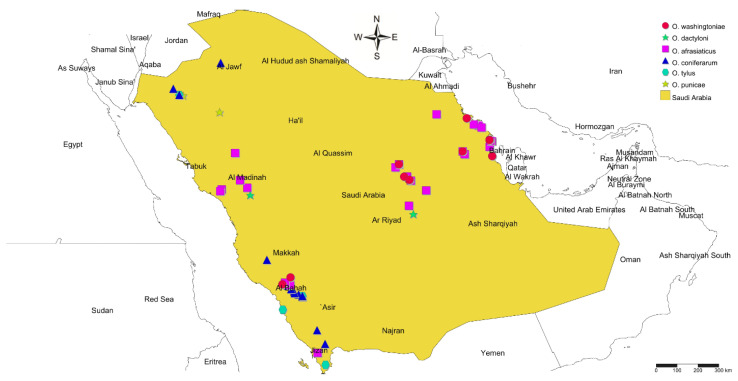
Geographical distribution of 61 spider mite samples of *Oligonychus afrasiaticus*, *O. coniferarum*, *O. dactyloni*, *O. punicae*, *O. tylus*, and *O. washingtoniae*, reported from various hosts and localities of 10 provinces in Saudi Arabia.

In total, 68 spider mite samples were collected from wild and cultivated vegetation in different seasons and localities from Mexico, Pakistan, SA, USA, and Yemen (Table S1; Figure 1). Out of 68 samples, 61 were collected from 10 Saudi provinces: Asir, Baha, Eastern Province, Jouf, Jizan, Makkah, Madinah, Qassim, Riyadh, and Tabuk (Table S1; Figure 2). The Saudi mite samples were collected between 2018 and 2021, except for one sample (sample voucher number/SVN: 39; Table S1), which was collected in 2013 from SA, and was claimed as *O. pratensis* [23]. The remaining 7 *Oligonychus* samples were received from 4 other countries: 1 from Mexico (SVN: 53), 1 from USA (SVN: 40), 2 from Yemen (SVN: 49 and 50), and 3 from Pakistan (SVN: 51, 66, and 67) (Table S1; Figure 1). Almost 2/3 of the mite samples had only female specimens (Table S1). Each sample’s collection details were recorded, e.g., sample voucher number (SVN), collection date, locality, host plant, GPS coordinates, collector name, etc. (Table S1). The voucher specimens of male/female representing each collected sample were deposited at the King Saud University Museum of Arthropods (Acarology section), Department of Plant Protection, College of Food and Agriculture Sciences, King Saud University (KSU), Riyadh, SA.

### 2.2. Mites Identification Based on Morphological Characters

Mite samples of *O. coniferarum*, *O. dactyloni*, *O. punicae*, *O. pratensis*, *O. tylus*, and *O. washingtoniae* were collected from SA and morphologically identified by Alatawi and Kamran [23]; Mushtaq, Kamran, and Alatawi [21]; and Mushtaq, Kamran, Saleh, and Alatawi [7]. The samples received from Mexico (SVN: 53, *O. perseae* Tuttle et al.), USA (SVN: 40, *O. pratensis*), and Yemen (SVN: 49 and 50, *O. afrasiaticus*) were initially identified/labelled and sent to us by our colleagues [24,25] at our request. Mite samples having male and female individuals were morphologically re-examined at the acarology laboratory (KSU) using the phase contrast microscope (BX51, Olympus, Tokyo, Japan) and identified till the species level following the taxonomic literature of the genus *Oligonychus* [3,26,27]. However, those 42 samples containing only female specimens were only identified up to the level of the genus *Oligonychus*, following Bolland, Gutierrez, and Flechtmann [26].

### 2.3. Molecular Analysis

#### 2.3.1. DNA Extraction and Amplification of ITS2/COI Regions

DNA extraction, from mite samples preserved in 99% ethanol, was carried out during the period from 2020 to 2021. The DNeasy mini kit (Qiagen^®^, Hilden, Germany) was used for DNA extraction from single adult mite females.

The concentration of genomic DNA solutions was assessed by the NanoDrop^TM^ One spectrophotometer (Thermofisher Scientific, Waltham, MA, USA). The extracted DNA samples were immediately stored at −20 °C after labelling with the appropriate field information.

The ITS2-rDNA region was amplified from mite samples using PCR primers, ITS2-forward (5′-GTCACATCTGTCTGAGAGTTGAGA-3′) and ITS2-reverse (5′-GTARCCTCACCTRMTCTGAGATC-3′) [16]. In addition, COI-forward primer (5′-TGATTTTTTGGTCACCCAGAAG-3′) and COI-reverse primer (5′-TACAGCTC CTATAGATA AAAC-3′) were also used to amplify the mtCOI region [12] (Table S1). The PCR reaction was performed with a total volume of 30 μL reaction containing 0.4 μL of each 10 μM primer, 15 μL 2× master mix (Molequle-On^®^, Auckland, New Zealand), ca 20 ng DNA, and appropriate volume of nuclease free water (Promega^®^, Madison, WI, USA). The PCR cycle conditions were as follows: (i) an initial denaturation cycle at 94 °C for 5 min; followed by (ii) 35 cycles of a denaturation step at 94 °C for 60 s, an annealing step for 90 s at 52 °C for ITS2, and 53 °C for COI and an extension step for 60 s at 72 °C; and (iii) a final extension step for 10 min at 72 °C. The obtained PCR products were run on 1.2% agarose gels in 1× TAE buffer. Gels were stained with acridine orange, observed, and picturized using the gel documentation BioDoc Analyze system (Uvitec, Cambridge, UK).

#### 2.3.2. DNA Sequencing and Analysis

The ITS2 and COI PCR products were purified and sequenced using the same primers at the Macrogen sequencing facility (Macrogen Inc., Seoul, Republic of Korea). The obtained sequences were cleaned and analyzed using BioEdit software [28]. The cleaned sequences were searched using BLASTn against the NCBI GenBank database. The homologous (sequence similarity within and among species were ˃98% and ˃85%, respectively) and closely related ITS2/COI sequences of all available *Oligonychus* species were retrieved from GenBank based on BLASTn results. The *Oligonychus* sequences retrieved from GenBank were aligned with their counterpart sequences obtained during the present study using the CLUSTALW multiple alignment tool in BioEdit. The retrieved GenBank ITS2/COI sequences were under the accession numbers: AB683673, OP363224, AB683666, AB683653, AB683662, AB683669, MW491838, AB683658, MN190324, KC009700, AB683660, AB683656, AB683677, LC341206, OP214345, AB683675, AB683655, MZ435900, MZ425483, X80866, AB683665, DQ656485, AB683681, AB683659, KU323485, X80865, GU329963, AB683664, and KC352302. In addition, some ITS2/COI sequences of unidentified *Oligonychus* species deposited from Australia (MF462136), India (MG429138, MG677944, MK386956, and OL830813), and USA (KP180428) were also retrieved from GenBank. All ITS2/COI sequences of *Oligonychus* species obtained during the present study were deposited in the NCBI-GenBank database (Table S1).

#### 2.3.3. Phylogenetic and Genetic Distances Analyses

Phylogenetic investigations were performed to assess the evolutionary relationships within and among various *Oligonychus* species using MEGA-X [29]. Phylogenetic trees were made using the neighbor-joining (NJ) method by the Tamura–Nei model [30]. The tree branch robustness was tested using the bootstrap analysis with 1000 replications [31]. Moreover, the pairwise *p*-distances (interspecific and intraspecific genetic divergence) were also calculated using MEGA-X.

## 3. Results

Based on the morphological examination of mite samples having male and female individuals, collected from different hosts and localities from Mexico, Pakistan, SA, USA, and Yemen, seven *Oligonychus* species viz. *O. afrasiaticus*, *O. coniferarum*, *O. dactyloni*, *O. pratensis*, *O. perseae*, *O. tylus,* and *O. washingtoniae* were recognized (Figure 1; Table S1). However, the samples containing only female specimens (Table S1) were recognized till the level of the genus *Oligonychus*.

**Figure 3:**
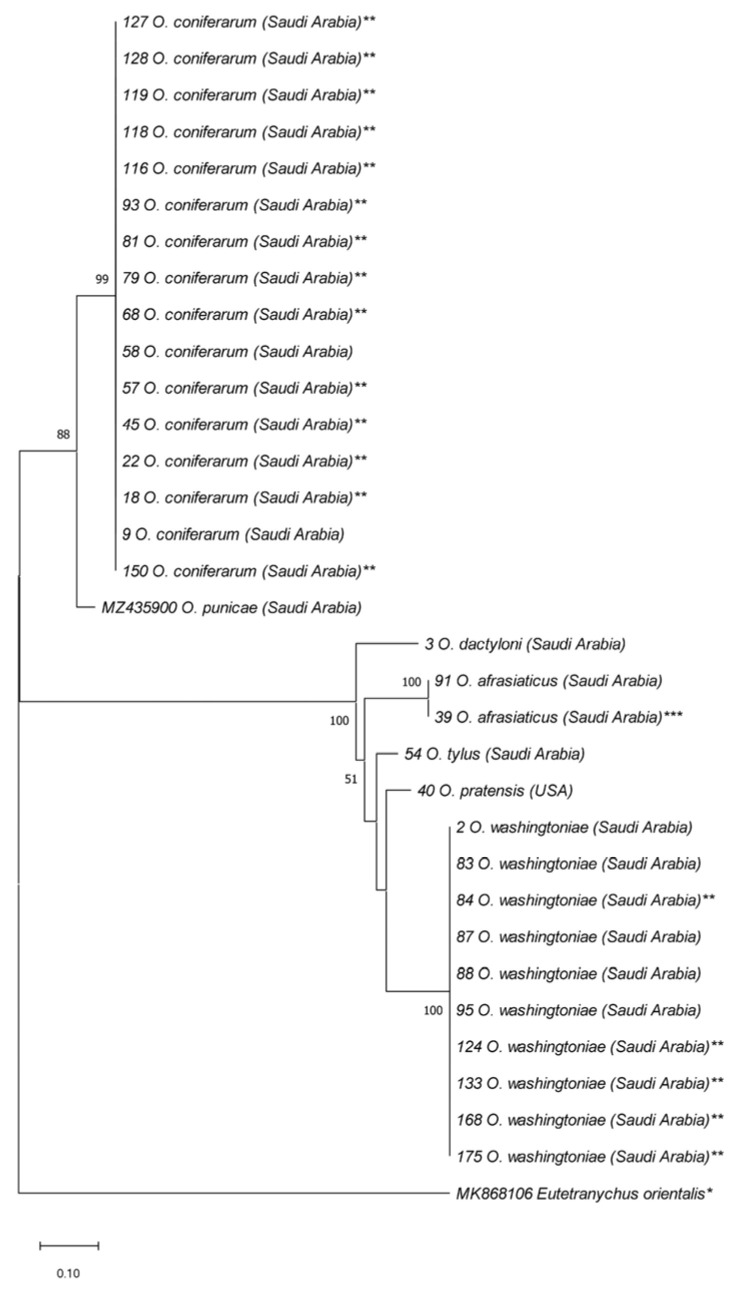
NJ phylogenetic tree based on 33 ITS2 sequences, representing different samples of seven *Oligonychus* species. Eutetranychus orientalis* was retrieved from GenBank and used as an outgroup taxon. A total of 30 sequences were obtained in the present study from different hosts and regions in Saudi Arabia and one from USA (** including 19 samples that have not been identified morphologically till species due to the absence of males, and *** one sample that was previously claimed as *O. pratensis* from Saudi Arabia) [23]. One sequence of *O. punicae* was retrieved from GenBank, previously reported from Saudi Arabia [7]. Numbers on tree branches are bootstrap values obtained from 1000 replicates.

**Figure 4:**
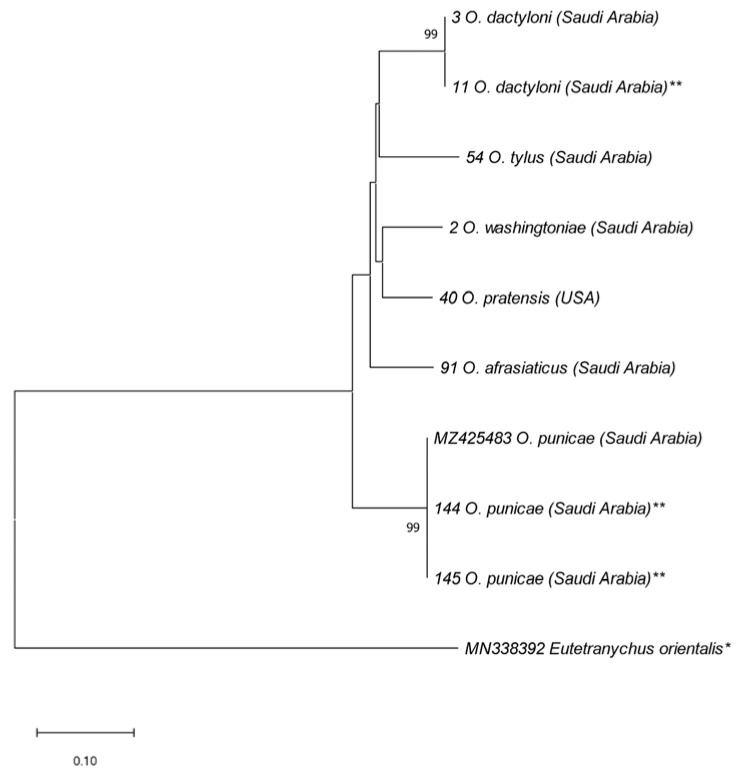
NJ phylogenetic tree based on 10 COI sequences, representing different samples of six *Oligonychus* species. Eutetranychus orientalis* was retrieved from GenBank and used as an outgroup taxon. A total of eight sequences were obtained in the present study from different hosts and regions in Saudi Arabia, and one from USA (** including samples that have not been identified morphologically till species due to the absence of males). One sequence of *O. punicae* was retrieved from GenBank, previously reported from Saudi Arabia [7]. Numbers on tree branches are bootstrap values obtained from 1000 replicates.

**Figure 5:**
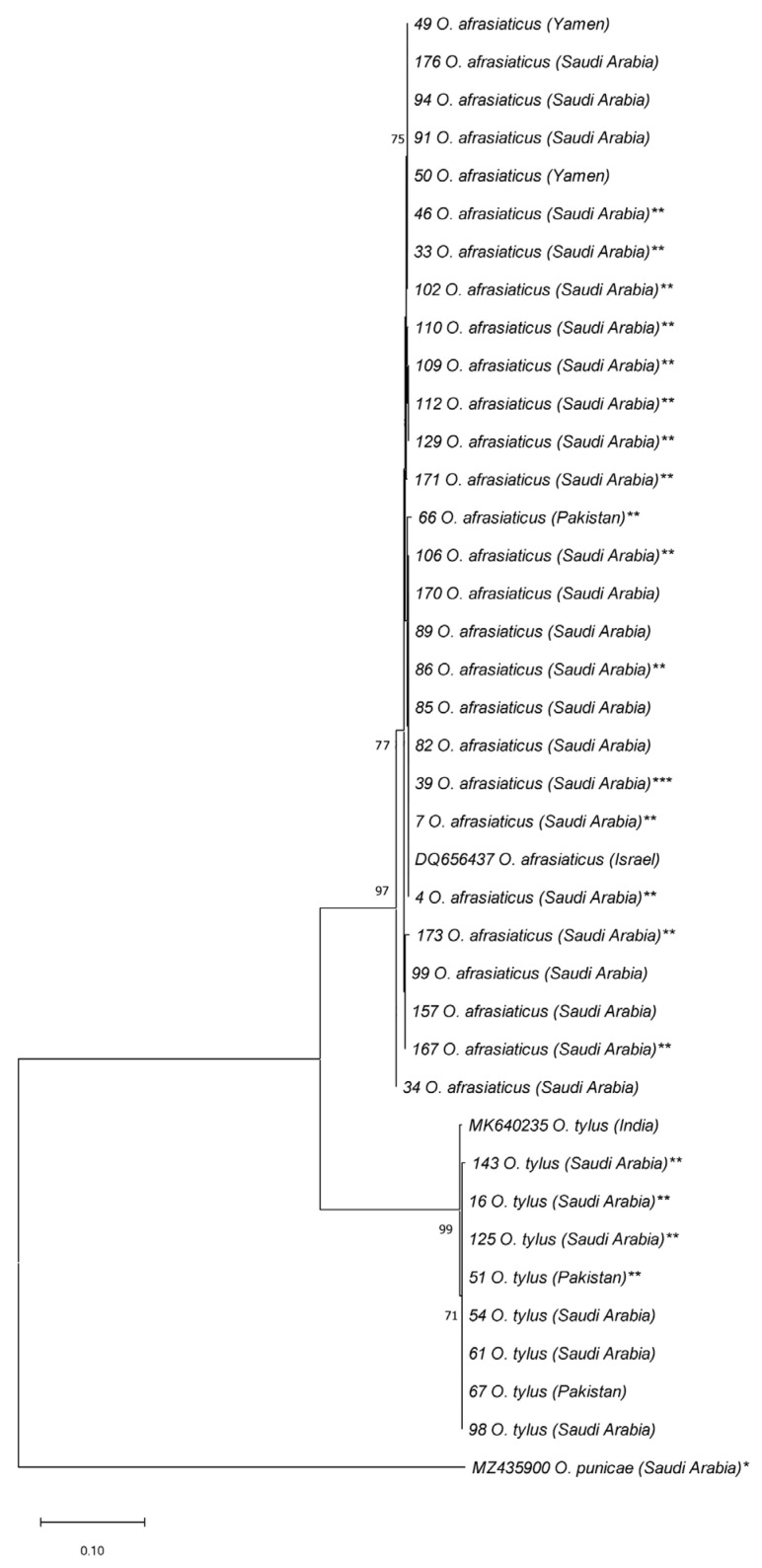
NJ tree based on 39 ITS2 sequences, representing different populations of the two closely related *Oligonychus* species (*O. afrasiaticus* and *O. tylus*) and one distantly related species, *O. punicae* (* was retrieved from GenBank and used as an outgroup taxon). A total of 31 sequences were obtained in the present study from different hosts and regions in Saudi Arabia, three from Pakistan, and two from Yemen. In total, 19 samples ** have not been morphologically identified till species level due to the absence of males, and *** one sample previously claimed as *O. pratensis* from Saudi Arabia [23]. The sequences of *O. afrasiaticus* from Israel and *O. tylus* from India were retrieved from GenBank. Numbers on tree branches are bootstrap values obtained from 1000 replicates.

**Figure 6:**
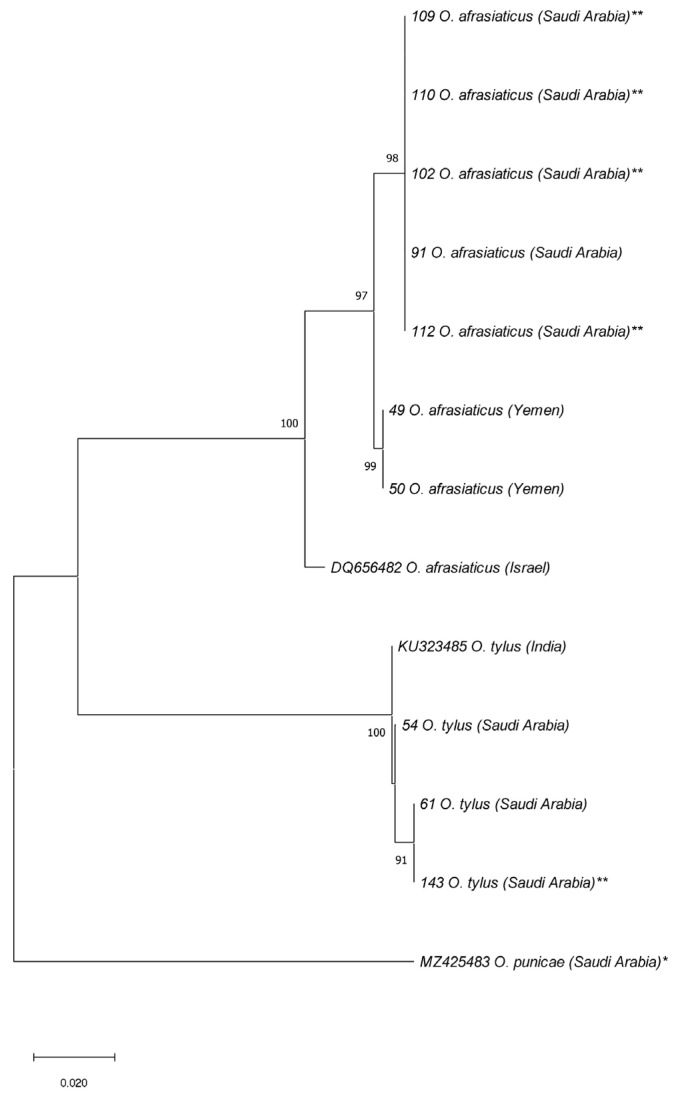
NJ phylogenetic tree based on 13 COI sequences, representing different populations of two closely related *Oligonychus* species (*O. afrasiaticus* and *O. tylus*) and one distantly related species, *O. punicae* (* was retrieved from GenBank and used as an outgroup taxon). A total of eight sequences were obtained in the present study from different hosts and regions in Saudi Arabia, and two from Yemen (** including samples that have not been identified morphologically till species due to the absence of males). The sequences of *O. afrasiaticus* from Israel and *O. tylus* from India were retrieved from GenBank. Numbers on tree branches are bootstrap values obtained from 1000 replicates.

**Figure 7:**
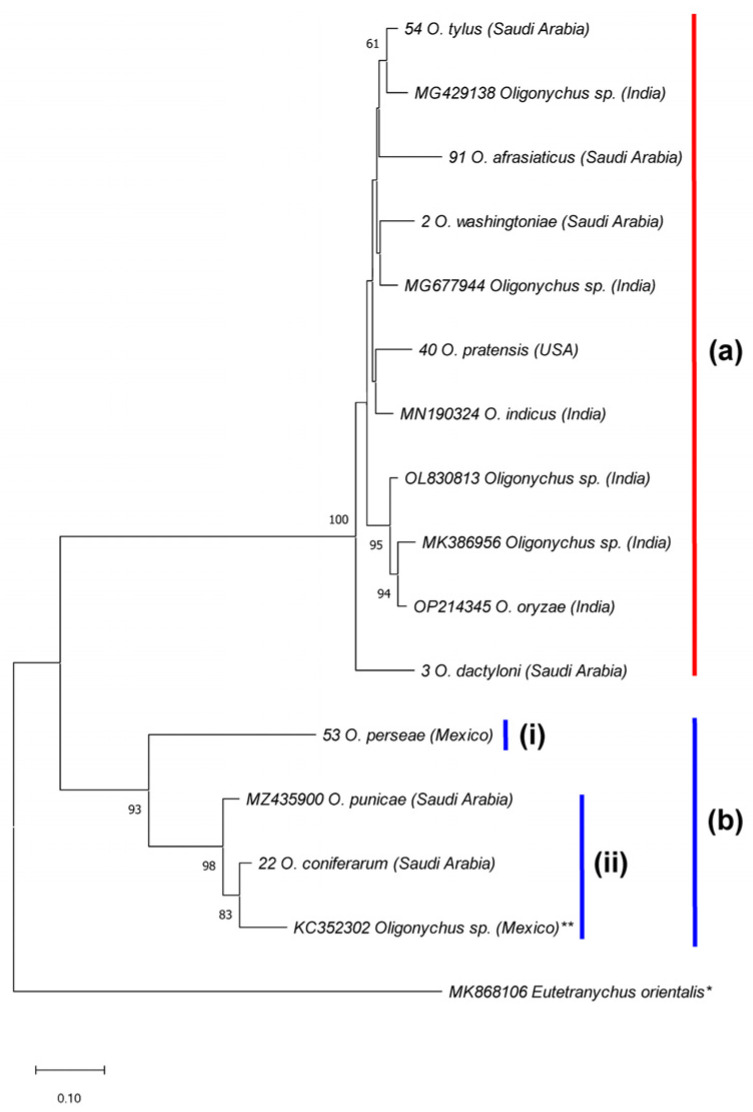
NJ phylogenetic tree based on 16 ITS2 sequences, representing 15 *Oligonychus* species. Eutetranychus orientalis* was used as an outgroup taxon. A total of 11 *Oligonychus* species belong to the subgenus (**a**) *Reckiella*, whereas four *Oligonychus* species (** including a cryptic *Oligonychus* sp., previously claimed as *O. punicae* from Mexico) [7] belong to the species groups (**b**-**i**) *peruvianus* and (**b**-**ii**) *coffeae* of the subgenus *Oligonychus* [3]. Numbers on tree branches are bootstrap values obtained from 1000 replicates.

At the molecular level, out of the 68 samples, 65 ITS2 amplicons were successfully amplified and sequenced. The length of the cleaned ITS2 sequences without ITS2 primers ranged from 405 to 539 bp (Table S1). However, the length of the 16 selected COI sequences obtained during this study was 410 bp (Table S1). Based on the ITS2 and COI data, the taxonomic identity of all 68 spider mite samples was successfully confirmed, which comprised eight *Oligonychus* species, namely *O. afrasiaticus*, *O. coniferarum*, *O. dactyloni*, *O. punicae*, *O. perseae*, *O. pratensis*, *O. tylus,* and *O. washingtoniae* (Figure 3, Figure 4, Figure 5, Figure 6 and Figure 7; Tables S1–S6). The actual taxonomic identity of the previously misidentified *O. pratensis* sample (SVN: 39; Table S1) that lacked male specimens was recognized as *O. afrasiaticus* (Figure 3; Table S2).

All the ITS2 and/or COI sequences of *O. coniferarum*, *O. dactyloni*, *O. punicae*, and *O. washingtoniae*, representing different samples from SA were assigned to a single haplotype (Tables S2 and S3; Figure 3 and Figure 4). Furthermore, the 29 ITS2 sequences of *O. afrasiaticus* separated into nine haplotypes: H1 (Israel and SA), H2 (SA and Yemen), H3 (SA), H4 (SA), H5 (SA), H6 (SA), H7 (Pakistan), H8 (SA), and H9 (SA) (Figure 5; Table S4). The nine ITS2 sequences of *O. tylus* were separated into four haplotypes: H1 (India), H2 (Pakistan and SA), H3 (SA), and H4 (SA) (Figure 5; Table S4). Likewise, the COI sequences of *O. afrasiaticus* and *O. tylus* were further separated into three and two haplotypes, respectively (Figure 6; Table S5). The three COI haplotypes of *O. afrasiaticus* were H1 (Israel), H2 (SA), and H3 (Yemen) (Figure 6; Table S5). Whereas the two COI haplotypes of *O. tylus* were H1 (India and SA) and H2 (SA) (Figure 6; Table S5).

The estimated pairwise *p*-distances for the ITS2 sequences showed that interspecific genetic divergence in various tested *Oligonychus* species ranged from 0.037 to 0.511 (3.7% to 51.1%; Tables S2 and S6), and intraspecific divergence ranged from 0.000 to 0.012 (0% to 1.2%; Table S4). The pairwise *p*-distances of COI sequences among various *Oligonychus* species showed interspecific divergence ranging from 0.032 to 0.181 (3.2% to 18.1%; Tables S3 and S7); whereas, the intraspecific divergence within same species ranged from 0.000 to 0.029 (0% to 2.9%; Table S5). Exceptionally, three COI sequences retrieved from the GenBank (AB683664 from Japan; GU329963 from China, and X80865 from France), representing three populations of *O. ununguis*, showed high intraspecific genetic divergence (6.5% to 11%; Table S7), suggesting *O. ununguis* is a species complex.

According to the ITS2-based NJ phylogenetic tree, the five species *O. afrasiaticus*, *O. dactyloni*, *O. pratensis*, *O. tylus*, and *O. washingtoniae* having upturned male aedeagus and belonging to the subgenus *Reckiella*, clustered as a monophyletic clade with 100% bootstrap value (Figure 3). However, *O. coniferarum* and *O. punicae* having downturned male aedeagus and belonging to the subgenus *Oligonychus*, formed a separate monophyletic clade with 88% bootstrap value (Figure 3). Similarly, in the COI-based NJ tree, the clade of the subgenus *Reckiella* and the subgenus *Oligonychus* grouped separately with monophyletic clades (Figure 4).

**Figure 8:**
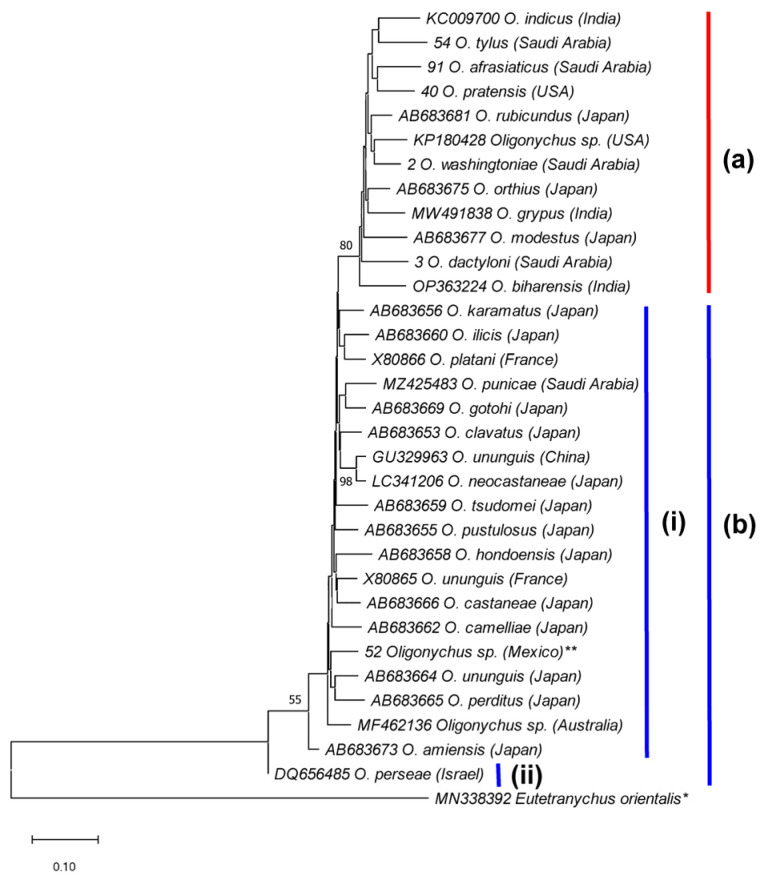
NJ phylogenetic tree based on 33 COI sequences, representing 32 *Oligonychus* species. Eutetranychus orientalis* was used as an outgroup taxon. A total of 12 *Oligonychus* species belong to the subgenus (**a**) *Reckiella*, whereas 18 *Oligonychus* species (** including a cryptic *Oligonychus* sp., previously claimed as *O. punicae* from Mexico) [7] belong to the species groups (**b**-**i**) *coffeae* and (**b**-**ii**) *peruvianus* of the subgenus *Oligonychus* [3]. Numbers on tree branches are bootstrap values obtained from 1000 replicates.

Moreover, the *Oligonychus* species of the subgenus *Reckiella* and species of the subgenus *Oligonychus* clustered separately into two monophyletic clades with 100% and 93% bootstrap values, respectively (Figure 7). In addition, the subgenus *Oligonychus* clade (supported with 93% bootstrap value) is further divided into two sub-clades, the b-i sub-clade, representing the *peruvianus* species group (female with either eight or nine tactile setae on tibia I), and the b-ii sub-clade representing the *coffeae* species group (female with either five, six, or seven tactile setae on tibia I) (Figure 7). Similarly, *Oligonychus* species of the subgenus *Reckiella* (Figure 8, clade a), and species of the subgenus *Oligonychus* (Figure 8, clade b) grouped separately into two monophyletic clades.

## 4. Discussion

In the present study, the ITS2 and COI molecular data successfully confirmed the species identity of eight *Oligonychus* species, namely, *O. afrasiaticus*, *O. coniferarum*, *O. dactyloni*, *O. punicae*, *O. pratensis*, *O. perseae*, *O. tylus*, and *O. washingtoniae*, representing 68 different mite samples collected from various hosts and localities in five countries. The genetic divergence is usually higher interspecifically than intraspecifically [7,14,16]. The ranges of interspecific genetic divergences obtained from ITS2 (3.7% to 51.1%), and COI (3.2% to 18.1%) supported the distinction of the eight *Oligonychus* species. However, the intraspecific genetic divergences ranged between 0% and 1.2% for ITS2 and 0% and 2.9% for COI. The obtained interspecific and intraspecific genetic divergences are aligned with the previous findings on tetranychid species [7,14,16,18,20,32,33], as well as other mites [34,35]. In various tetranychid species, including three *Oligonychus* species of *O. afrasiaticus*, *O. perseae*, and *O. mangiferus* (=*O. punicae*), the ITS2-based interspecific nucleotide divergence ranged from 4.4% to 54.8% [16]. Moreover, the interspecific divergence for ITS2 detected among four closely related *Oligonychus* species belonging to the subgenus *Oligonychus* ranged from 11.5% to 18.8% [7]. Similarly, the COI-based interspecific nucleotide divergences ranging from 5.4% to 18.3%, were detected among various *Oligonychus* species [7,14].

The absence of male specimens can lead to the creation of doubtful *Oligonychus* species, especially in the absence of integrative taxonomic approaches [3]. For example, *O. changi* (Tseng), *O. jiangxiensis* (Ma and Yuan), *O. longus* (Chaudhri, Akbar, and Rasool), and *O. pongami* (Sivakumar and Kunchithapatham) are suggested as *species inquirendae*, which were described without males [3,11,36,37,38]. In the present study, the molecular data revealed the correct species identity of 42 mite samples that lacked male specimens. Based on the low intraspecific nucleotide divergence (0% to 1.2%, ITS2; 0% to 2.9%, COI) and phylogenetic analyses, 16 samples of *O. afrasiaticus*, 14 samples of *O. coniferarum*, 1 sample of *O. dactyloni*, 2 samples of *O. punicae*, 4 samples of *O. tylus*, and 5 samples of *O. washingtoniae* were successfully recognized till the level of species. Additionally, the *Oligonychus* Saudi population, previously claimed *O. pratensis* [23], was correctly recognized as *O. afrasiaticus*. The absence of males in the *Oligonychus* population collected from grasses in the Baha region of SA led to the misidentification as *O. pratensis* [23]. Indeed, while conducting this study, we have not found any population of *O. pratensis* from SA. When we compared all the Saudi samples of *Oligonychus* belonging to the subgenus *Reckiella* with the Californian sample of *O. pratensis* collected from the USA (the country of its type locality), high genetic divergence either based on ITS2 (>11%) or COI (>8%), was shown, which is in accordance with separate species in previous molecular studies on tetranychid mites [7,14,16].

The wide distribution and polyphagous behavior of a spider mite species may affect its genetic structure [17,39]. The current study also revealed high genetic variations (number of haplotypes) in two widely distributed oligophagous species *O. afrasiaticus* and *O. tylus* collected/analyzed from India, Israel, Pakistan, SA, and Yemen. Nine ITS2 and three COI haplotypes have been identified in the date palm mite *O. afrasiaticus*. Out of the nine ITS2 haplotypes, representing seven hosts and four countries, six (H3, H4, H5, H6, H8, and H9) were only recovered from SA, one from both SA and Israel (H1), one from SA and Yemen (H2), and one (H7) from Pakistan. The geographic isolation may explain the distinction between the Middle Eastern and Pakistani ITS2 haplotypes of *O. afrasiaticus* [40]. Geographic isolation was a key factor in generating genetic variations among various populations of the citrus red mite *Panonychus citri* (McGregor) [41]. Similarly, *O. tylus* showed four ITS2 haplotypes representing nine localities and two COI haplotypes representing four localities, recovered from seven hosts and two countries. In addition to geographic distribution, host plants can increase genetic variations, as exhibited by the polyphagous spider mite species *Eutetranychus orientalis* (Klein) [17]. The oligophagous *O. coniferarum* inhabiting different species of conifers and the monophagous *O. washingtoniae* inhabiting *W. filifera*, did not show any genetic variations among their populations collected from different localities of SA.

The phylogenetic trees constructed using ITS2 sequences of 15 *Oligonychus* species and COI sequences of 30 *Oligonychus* species confirmed the morphological-based subdivision of the genus *Oligonychus* into two subgenera and four species groups [3]. The phylogenetic trees showed the monophyly nature of the two subgenera of *Oligonychus*: *Reckiella* and *Oligonychus*. These results are in agreement with previous findings either based on DNA-Sequence data of ITS2, COI, and 28S regions [14,16,32,42], or RNA-Sequence data [43], which separated *Oligonychus* species into two clades: a group of species with upturned (the subgenus *Reckiella*) and others with the downturned direction of male aedeagus (the subgenus *Oligonychus*). Further, in ITS2/COI-based phylogenetic trees, species belonging to the *coffeae* species group were clustered distantly from *O. perseae* of the *peruvianus* species group. It confirmed the morphological-based subdivision of the subgenus *Oligonychus* into two species groups based on a female character, i.e., the number of tactile setae on tibia I [3].

In addition, the molecular analysis of COI sequences of *O. ununguis* available on GenBank showed that the three cryptic species are hidden under the *ununguis* complex. The Japanese (Accession no: AB683664), Chinese (Accession no: GU329963), and French (Accession no: X80865) sequences of *O. ununguis* showed high genetic divergences (6.5% to 11%) from one another. Such high nucleotide divergences fall within the range of interspecific genetic divergence, as observed in the present (3.2% to 18.1%) and previous studies (5.4% to 18.3%) on *Oligonychus* species [7,14]. Similarly, the two GenBank ITS2 sequences of *O. ununguis* from China and Korea were previously highlighted as doubtful [7].

Some ITS2 (Accession no: MG429138, MG677944, OL830813, MK386956, India) and COI (KP180428, USA; MF462136, Australia) sequences available on GenBank identified to the genus *Oligonychus* level. Molecular data could not assign them to any of the known *Oligonychus* species. The high genetic divergences of ITS2 (4.4% to 10.4%) and COI (12.6%) clearly indicate their taxonomic identity as separate *Oligonychus* species. However, it is strongly recommended that the specimens/vouchers of these sequences need to be morphologically and molecularly re-characterized to know the actual global fauna of the genus *Oligonychus*.

## 5. Conclusions

In conclusion, the molecular data of ITS2 and COI successfully confirmed the taxonomic identity of 68 spider mite samples collected either with or without male specimens, representing eight *Oligonychus* species from Mexico, Pakistan, SA, USA, and Yemen. The phylogenetic analyses of various *Oligonychus* species also validated the subdivision of the genus *Oligonychus* into subgenera and species groups. The present study highlights the importance of integrated taxonomic approaches, e.g., the combination of morphological and molecular data, to confirm the identity of closely related *Oligonychus* species, especially in the absence of key morphological characters or individuals. Additionally, molecular data showed that the *O. ununguis* sequences available on the NCBI-GenBank database might be taxonomically misidentified and need further work. Overall, a taxonomic revision is compulsory to resolve the global issue of species complexes by adopting integrative taxonomic approaches.

## Data Availability

All necessary data is provided in this paper.

## Supplementary Materials

The following supporting information can be downloaded at: https://www.mdpi.com/article/10.3390/insects14020192/s1, Table S1: Geographical distribution, host plant, collection details, and ITS2/COI fragment size of all spider mite samples of seven *Oligonychus* species viz. *O. afrasiaticus*, *O. coniferarum*, *O. dactyloni*, *O. punicae*, *O. pratensis*, *O. tylus*, and *O. washingtoniae*, collected from Pakistan, Saudi Arabia, USA, & Yamen, and analyzed in the present study. Table S2: Genetic divergence (pairwise p-distance) based on 33 ITS2 partial sequences, either obtained in the present study or retrieved from GenBank, among seven *Oligonychus* species (Acari: Prostigmata: Tetranychidae), collected/analyzed from Saudi Arabia and the USA. Table S3: Genetic divergence (pairwise p-distance) based on nine COI partial sequences, either obtained in the present study or retrieved from GenBank, among six *Oligonychus* species (Acari: Prostigmata: Tetranychidae), collected/analyzed from Saudi Arabia and the USA. Table S4: Genetic divergence (pairwise p-distance) based on 38 ITS2 partial sequences, either obtained in the present study or retrieved from GenBank, showing variations among various populations of *Oligonychus afrasiaticus* and *O. tylus* (Acari: Prostigmata: Tetranychidae), collected/analyzed from Israel, India, Pakistan, Saudi Arabia, and Yemen. Table S5: Genetic divergence (pairwise p-distance) based on 12 COI partial sequences, either obtained in the present study or retrieved from GenBank, showing variations among various populations of *Oligonychus afrasiaticus* and *O. tylus* (Acari: Prostigmata: Tetranychidae), collected/analyzed from Israel, India, Saudi Arabia, and Yemen. Table S6: Genetic divergence (pairwise p-distance) based on ITS2 sequences among 15 *Oligonychus* species (Acari: Prostigmata: Tetranychidae), either obtained in the present study or retrieved from GenBank, analyzed from India, Mexico, Saudi Arabia, and the USA. Table S7: Genetic divergence (pairwise p-distance) based on COI sequences among 32 *Oligonychus* species (Acari: Prostigmata: Tetranychidae), either obtained in the present study or retrieved from GenBank. References [7,23] are cited in the Supplementary Materials.
