# Supplementary material for: Molecular-Based Taxonomic Inferences of Some Spider Mite Species of the Genus Oligonychus Berlese (Acari, Prostigmata, Tetranychidae)
Source: Insects. 2023 Feb 15;14(2):192. doi: 10.3390/insects14020192 (PMC9959262; doi:10.3390/insects14020192)
Supplement: Supplementary file 1 [file insects-14-00192-s001.zip › insects-2186525-supplementary.pdf]

Molecular-Based Taxonomic Inference of Some Spider Mite Species of the Genus *Oligonychus* Berlese (Acari, Prostigmata, Tetranychidae)

Hafiz Muhammad Saqib Mushtaq [1], Amgad A. Saleh [2], Muhammad Kamran [1], Fahad Jaber Alatawi [1,*]

[1] Acarology Research Laboratory, Department of Plant Protection, College of Food and Agriculture Sciences, King Saud University, P.O. Box No. 2460, Riyadh 11451, Saudi Arabia
[2] Plant Pathology Laboratory, Department of Plant Protection, College of Food and Agriculture Sciences, King Saud University, P.O. Box No. 2460, Riyadh 11451, Saudi Arabia
***** Correspondence: falatawi@ksu.edu.sa

Number of supplementary tables: 7

Table S1. Geographical distribution, host plant, collection details, and ITS2/COI fragment size of all spider mite samples of seven *Oligonychus* species viz. *O. afrasiaticus*, *O. coniferarum*, *O. dactyloni*, *O. punicae*, *O. pratensis*, *O. tylus*, and *O. washingtoniae*, collected from Pakistan, Saudi Arabia, USA, & Yamen, and analyzed in the present study.

Sample voucher no. (SVN)	Species	Collection date	Locality, Province, Country	Host Plant	Collector name (Reference)	GPS	GenBank accession numbers		ITS2 Fragment Size (bp)	COI Fragment Size (bp)
							ITS2	COI		
2	*O. washingtoniae*	02 Jun 2018	Diriyah, Riyadh, Saudi Arabia	*Washingtonia filifera* (Lindl.) H. Wendl. (Arecaceae)	HMS Mushtaq	24°44'48.7"N, 46°31'37.0"E	OQ185439	OQ180838	483	410
3	*O. dactyloni*	19 May 2018	Haylat Radi al Baham, Madinah, Saudi Arabia	Grasses (Poaceae)	HMS Mushtaq, JH Mirza & M Kamran	24°05'19.3"N 39°40'06.9"E	OQ185449	OQ180840	405	410
4*	*O. afrasiaticus*	07 Aug 2018	KSU, Riyadh, Saudi Arabia	Grasses	HMS Mushtaq	24°42'59.2"N 46°36'42.8"E	OQ185402	N/A	517	N/A
7*	*O. afrasiaticus*	08 May 2018	Hariq, Riyadh, Saudi Arabia	*Phoenix dactylifera* L. (Arecaceae)	HMS Mushtaq, JS Basahih & M Kamran	23°37'50.6"N 46°31'56.5"E	OQ185404	N/A	517	N/A
9	*O. coniferarum*	13 Apr 2018	Taif, Makkah, Saudi Arabia	*Thuja* sp. (Cupressaceae)	HMS Mushtaq	21°17'23.5"N 40°23'18.5"E	OQ185386	N/A	460	N/A
11*	*O. dactyloni*	08 May 2018	Howtat Bani Tamim, Riyadh, Saudi Arabia	Grasses	HMS Mushtaq, JS Basahih & M Kamran	23°15'50.3"N 46°43'18.6"E	N/A	OQ180841	N/A	410
16*	*O. tylus*	12 May 2018	Kharabah, Jizan, Saudi Arabia	Grasses	HMS Mushtaq, JS Basahih & M Kamran	16°44'51.8"N 42°55'33.8"E	OQ185437	N/A	515	N/A
18*	*O. coniferarum*	24 May 2018	JADCO, Jouf, Saudi Arabia	*Thuja* sp.	HMS Mushtaq, JH Mirza & M Kamran	29°48'44.5"N 38°23'22.6"E	OQ185387	N/A	483	N/A
22*	*O. coniferarum*	23 May 2018	Cultivated Area, Tabuk, Saudi Arabia	*Thuja* sp.	HMS Mushtaq, JH Mirza & M Kamran	28°25'22.7"N 36°36'02.7"E	OQ185388	N/A	482	N/A
33*	*O. afrasiaticus*	21 Apr 2018	Qatif, Eastern Province, Saudi Arabia	*Sorghum halepense* (L.) Pers. (Poaceae)	JS Basahih, JH Mirza & M Kamran	27°35'16.1"N 47°43'30.3"E	OQ185412	N/A	520	N/A
34	*O. afrasiaticus*	20 Apr 2018	Qaryat AlUlya, Eastern Province, Saudi Arabia	Grasses	JS Basahih, JH Mirza & M Kamran	27°35'16.1"N 47°43'30.3"E	OQ185423	N/A	492	N/A
39*[1]	*O. afrasiaticus*	24 Apr 2013	Allaghamees, Baha, Saudi Arabia	Grasses	M Kamran	20°11'52.5"N 41°23'35.8"E	OQ185405	N/A	517	N/A
40	*O. pratensis*	Spring (2015)	Hadley's Date Gardens, California, USA	*P. dactylifera*	F Ganjisaffar	33 38'25"N, 116 12'11"W	OQ185450	OQ180839	530	410
45*	*O. coniferarum*	22 May 2018	Astra farms, Tabuk, Saudi Arabia	*Thuja* sp.	HMS Mushtaq, JH Mirza & M Kamran	28°41'32.0"N 36°20'31.1"E	OQ185389	N/A	483	N/A
46*	*O. afrasiaticus*	11 May 2018	Malha, Jizan, Saudi Arabia	*S. bicolor*	HMS Mushtaq, JS Basahih & M Kamran	17°16'02.3"N 42°34'46.9"E	OQ185413	N/A	520	N/A

49	*O. afrasiaticus*	N/A	Seiyun, Hadhramout, Yemen	*P. dactylifera*	JH Basahih	15°54'58.5"N 48°46'53.0"E	OQ185414	OQ180833	515	410
50	*O. afrasiaticus*	27 May 2019	Hisn ‘Idan, Hadhramout, Yemen	*P. dactylifera*	JH Basahih	15°20'20.8"N 48°46'53.0"E	OQ185415	OQ180834	520	410
51*	*O. tylus*	08 Aug 2019	BZU, Punjab, Pakistan	*Z. mays*	N Abbas	30°16'04.5"N 71°30'03.7"E	OQ185431	N/A	539	N/A
53	*O. perseae*	09 Oct 2019	Chiautla, Estado de México, Mexico	*Alnus jorulensis* Kunth (Betulaceae)	MT Santillán-Galicia	19°32'33.1"N 98°52'33.1"W	OQ185403	N/A	470	N/A
54	*O. tylus*	01 Nov 2019	Qunfudhah, Makkah, Saudi Arabia	*Hyphaene thebaica* (L.) Mart.	HMS Mushtaq, & M Kamran	19°08'01.3"N 41°04'33.3"E	OQ185432	OQ180835	517	410
57*	*O. coniferarum*	31 Oct 2019	KKU, Asir, Saudi Arabia	*Arucaria* sp. (Cupressaceae)	HMS Mushtaq, & M Kamran	18°14'51.3"N 42°33'28.0"E	OQ185390	N/A	483	N/A
58	*O. coniferarum*	28 Oct 2019	Baljuraishi, Baha, Saudi Arabia	*Arucaria* sp.	HMS Mushtaq, & M Kamran	19°55'27.9"N 41°33'42.5"E	OQ185391	N/A	483	N/A
61	*O. tylus*	29 Oct 2019	Bashayer, Asir, Saudi Arabia	*S. bicolor*	HMS Mushtaq, & M Kamran	19°43'26.3"N 41°55'30.8"E	OQ185433	OQ180836	517	410
66*	*O. afrasiaticus*	22 Aug 2019	Wan Bachran/Mianwali, Punjab, Pakistan	*Pennisetum glaucum* (L.) R.Br. (Poaceae)	EM Khan	32°24'23.1"N 71°43'29.6"E	OQ185428	N/A	517	N/A
67	*O. tylus*	22 Aug 2019	Wan Bachran/Mianwali, Punjab, Pakistan	*P. glaucum*	EM Khan	32°24'23.1"N 71°43'29.6"E	OQ185434	N/A	535	N/A
68*	*O. coniferarum*	28 Oct 2019	Wadi Shora, Baha, Saudi Arabia	*Thuja* sp.	HMS Mushtaq	19°47'36.6"N 41°45'45.3"E	OQ185392	N/A	460	N/A
79*	*O. coniferarum*	28 Oct 2019	Baljuraishi, Baha, Saudi Arabia	*Thuja* sp.	HMS Mushtaq & M Kamran	19°50'47.0"N 41°35'06.2"E	OQ185393	N/A	458	N/A
81*	*O. coniferarum*	29 Oct 2019	Ajbah, Asir, Saudi Arabia	*Thuja* sp.	HMS Mushtaq & M Kamran	19°41'27.0"N 41°55'37.0"E	OQ185394	N/A	482	N/A
82	*O. afrasiaticus*	18 Oct 2019	Khobar, Eastern Province, Saudi Arabia	*W. filifera*	HMS Mushtaq & M Kamran	26°25'07.1"N 50°07'14.5"E	OQ185406	N/A	517	N/A
83	*O. washingtoniae*	17 Oct 2019	Uqair beach, Eastern Province, Saudi Arabia	*W. filifera*	HMS Mushtaq & M Kamran	25°46'43.2"N 50°07'53.7"E	OQ185440	N/A	483	N/A
84*	*O. washingtoniae*	13 April 2019	Uyaynah, Riyadh, Saudi Arabia	*W. filifera*	HMS Mushtaq & M Kamran	24°53'13.9"N, 46°19'26.5"E	OQ185441	N/A	510	N/A
85	*O. afrasiaticus*	18 Oct 2019	Khobar, Eastern Province, Saudi Arabia	*P. dactylifera*	HMS Mushtaq	26°10'47.6"N 50°01'07.8"E	OQ185407	N/A	517	N/A
86*	*O. afrasiaticus*	13 April 2019	Jubaylah, Riyadh, Saudi Arabia	*W. filifera*	HMS Mushtaq	24°53'49.2"N, 46°26'54.3"E	OQ185408	N/A	517	N/A
87	*O. washingtoniae*	19 Oct 2019	Ras Al-Khair, Eastern Province, Saudi Arabia	*W. filifera*	HMS Mushtaq	27°24'53.6"N 49°01'56.1"E	OQ185442	N/A	510	N/A
88	*O. washingtoniae*	18 April 2019	Wadi Hanifah, Riyadh, Saudi Arabia	*W. filifera*	HMS Mushtaq	24°44'57.2"N 46°33'24.0"E	OQ185443	N/A	510	N/A
89	*O. afrasiaticus*	22 June 2019	KSU, Riyadh, Saudi Arabia	*P. dactylifera*	HMS Mushtaq	24°42'59.2"N 46°36'42.8"E	OQ185409	N/A	517	N/A
91	*O. afrasiaticus*	18 Oct 2019	Damam, Eastern Province, Saudi Arabia	Grasses	HMS Mushtaq & M Kamran	26°26'17.4"N 50°03'01.1"E	OQ185416	OQ180828	520	410
93*	*O. coniferarum*	29 Oct 2019	Bashayer, Asir, Saudi Arabia	*Pulicaria* sp. (Asteraceae)	HMS Mushtaq & M Kamran	19°43'26.3"N 41°55'30.8"E	OQ185395	N/A	483	N/A

94	*O. afrasiaticus*	10 Oct 2019	Jubail, Eastern Province, Saudi Arabia	*Cynodon dactylon* (L.) Pers. (Poaceae)	HMS Mushtaq & M Kamran	27°06'10.0"N 49°32'30.1"E	OQ185417	N/A	520	N/A
95	*O. washingtoniae*	18 Oct 2019	Damam, Eastern Province, Saudi Arabia	*W. filifera*	HMS Mushtaq & M Kamran	26°28'31.1"N 50°00'47.5"E	OQ185444	N/A	510	N/A
98	*O. tylus*	18 Oct 2019	Damam, Eastern Province, Saudi Arabia	Grasses	HMS Mushtaq & M Kamran	26°28'31.1"N 50°00'47.5"E	OQ185435	N/A	539	N/A
99	*O. afrasiaticus*	19 Oct 2019	Jubail, Eastern Province, Saudi Arabia	*P. dactylifera*	HMS Mushtaq & M Kamran	27°08'50.1"N 49°20'02.4"E	OQ185420	N/A	515	N/A
102*	*O. afrasiaticus*	19 Oct 2019	Jubail, Eastern Province, Saudi Arabia	Grasses	HMS Mushtaq & M Kamran	27°00'59.7"N 49°40'11.7"E	OQ185418	OQ180829	519	410
106	*O. afrasiaticus*	24 Aug 2020	Abyar ‘Ali, Madinah, Saudi Arabia	*P. dactylifera*	FJ Alatawi, JH Mirza & EM Khan	24°24'33.6"N 39°32'28.8"E	OQ185410	N/A	519	N/A
109*	*O. afrasiaticus*	25 Aug 2020	Buwat, Madinah, Saudi Arabia	*C. dactylon*	FJ Alatawi, JH Mirza & EM Khan	24°44'10.3"N 39°13'33.7"E	OQ185425	OQ180830	519	410
110*	*O. afrasiaticus*	26 Aug 2020	Yanbu Al Nakhal, Madinah, Saudi Arabia	*C. dactylon*	FJ Alatawi, JH Mirza & EM Khan	24°20'18.2"N 38°25'57.8"E	OQ185429	OQ180831	519	410
112*	*O. afrasiaticus*	26 Aug 2020	Mabaruck Village, Madinah, Saudi Arabia	Grasses	FJ Alatawi, JH Mirza & EM Khan	24°15'37.8"N 38°22'15.2"E	OQ185426	OQ180832	519	410
116*	*O. coniferarum*	10 Sep 2020	Wadi Zarq, Baha, Saudi Arabia	*Thuja* sp.	HMS Mushtaq, JH Mirza & HMS Ali	20°11'19.8"N 41°17'27.8"E	OQ185396	N/A	483	N/A
118*	*O. coniferarum*	10 Sep 2020	Raghdan Forest Park, Baha, Saudi Arabia	*Thuja* sp.	HMS Mushtaq, JH Mirza & HMS Ali	20°01'15.5"N 41°26'10.3"E	OQ185397	N/A	483	N/A
119*	*O. coniferarum*	10 Sep 2020	Musaylah, Baha, Saudi Arabia	*Thuja* sp.	HMS Mushtaq, JH Mirza & HMS Ali	20°10'42.4"N 41°17'04.7"E	OQ185398	N/A	483	N/A
124*	*O. washingtoniae*	11 Sep 2020	Hajrah, Baha, Saudi Arabia	*Washingtonia* sp.	HMS Mushtaq, JH Mirza & HMS Ali	20°13'49.4"N 41°03'32.8"E	OQ185445	N/A	510	N/A
125*	*O. tylus*	11 Sep 2020	Qura General Hospital, Baha, Saudi Arabia	Grasses	HMS Mushtaq, JH Mirza & HMS Ali	20°16'17.0"N 41°09'03.0"E	OQ185438	N/A	515	N/A
127*	*O. coniferarum*	11 Sep 2020	Doos, Baha, Saudi Arabia	*Arucaria* sp.	HMS Mushtaq, JH Mirza & HMS Ali	20°17'06.2"N 41°13'29.9"E	OQ185399	N/A	483	N/A
128*	*O. coniferarum*	11 Sep 2020	Doos, Baha, Saudi Arabia	*Thuja* sp.	HMS Mushtaq, JH Mirza & HMS Ali	20°17'06.2"N 41°13'29.9"E	OQ185400	N/A	483	N/A
129*	*O. afrasiaticus*	11 Sep 2020	Bani Omran Village, Baha, Saudi Arabia	*Pulicaria* sp.	HMS Mushtaq, JH Mirza & HMS Ali	20°18'11.0"N 41°10'23.1"E	OQ185427	N/A	518	N/A
133*	*O. washingtoniae*	12 Sep 2020	Mashoqa Park, Baha, Saudi Arabia	*Washingtonia* sp.	HMS Mushtaq, JH Mirza & HMS Ali	20°31'51.2"N, 41°24'36.9"E	OQ185446	N/A	510	N/A
143*	*O. tylus*	30 Sep 2020	Muruj, Tabuk, Saudi Arabia	Grasses	HMS Mushtaq, JH Mirza & EM Khan	28°25'11.0"N 36°36'10.8"E	OQ185436	OQ180837	516	410
144*	*O. punicae*	30 Sep 2020	Tabuk, Tabuk, Saudi Arabia	Unknown	HMS Mushtaq, JH Mirza & EM Khan	28°23'40.3"N 36°45'59.8"E	N/A	OQ180826	N/A	410
145*	*O. punicae*	01 Oct 2020	Tayma, Tabuk, Saudi Arabia	Unknown	HMS Mushtaq, JH Mirza & EM Khan	27°40'31.0"N 38°20'35.7"E	N/A	OQ180827	N/A	410
150*	*O. coniferarum*	09 Oct 2020	Jabal AlQahar, Jizan, Saudi Arabia	*Thuja* sp.	HMS Mushtaq, JH Mirza & EM Khan	17°39'13.1"N 42°54'16.7"E	OQ185401	N/A	483	N/A
157	*O. afrasiaticus*	22 Oct 2020	Urayarah, Eastern Province, Saudi Arabia	*P. dactylifera*	JH Mirza, HMS Sajid, N Elgoni & M Waleed	25°58'54.2"N 48°51'02.1"E	OQ185421	N/A	514	N/A

167*	*O. afrasiaticus*	22 Oct 2020	Um Alerrad, Eastern Province, Saudi Arabia	*Washingtonia* sp.	JH Mirza, HMS Sajid, N Elgoni & M Waleed	25°51'39.5"N 48°56'18.9"E	OQ185422	N/A	514	N/A
168*	*O. washingtoniae*	22 Oct 2020	Urayarah, Eastern Province, Saudi Arabia	*Washingtonia* sp.	JH Mirza, HMS Sajid, N Elgoni & M Waleed	25°58'54.2"N 48°51'02.1"E	OQ185447	N/A	510	N/A
170	*O. afrasiaticus*	31 Oct 2020	Bir, Riyadh, Saudi Arabia	*P. dactylifera*	HMS Mushtaq, EM Khan & N Elgoni	25°17'25.8"N 45°56'45.7"E	OQ185411	N/A	516	N/A
171*	*O. afrasiaticus*	07 Nov 2020	Kharj, Riyadh, Saudi Arabia	Grasses	HMS Mushtaq, EM Khan & N Elgoni	24°17'33.4"N 47°16'42.9"E	OQ185424	N/A	518	N/A
173*	*O. afrasiaticus*	31 Oct 2020	Hasi, Riyadh, Saudi Arabia	Unknown	HMS Mushtaq, EM Khan & N Elgoni	25°25'13.8"N 46°05'54.9"E	OQ185430	N/A	516	N/A
175*	*O. washingtoniae* .	31 Oct 2020	Hasi, Riyadh, Saudi Arabia	*Washingtonia* sp.	HMS Mushtaq, EM Khan & N Elgoni	25°25'13.8"N 46°05'54.9"E	OQ185448	N/A	510	N/A
176*	*O. afrasiaticus*	28 Mar 2021	Uyaynah, Madinah, Saudi Arabia	Grasses	HMS Mushtaq, M Kamran	25°54'38.3"N 39°01'00.0"E	OQ185419	N/A	519	N/A

* These 42 samples did not contain male specimens and were morphologically identified till the level of the genus *Oligonychus*. However, their molecular analysis confirmed their species' identity.

[1] It represents the sample of *O. afrasiaticus*, containing only female/nymph specimens and previously claimed as *O. pratensis* from Saudi Arabia [1].

Table S2. Genetic divergence (pairwise p-distance) based on 33 ITS2 partial sequences, either obtained in the present study or retrieved from GenBank, among seven *Oligonychus* species (Acari: Prostigmata: Tetranychidae), collected/analyzed from Saudi Arabia and the USA.

No.	***Oligonychus* species**	1	2	3	4	5	6	7
**1**	*O. punicae* (MZ435900, Saudi Arabia)	0.000						
**2**	*O. coniferarum**	0.093	0.000					
**3**	*O. afrasiaticus***	0.478	0.488	0.000 – 0.012				
**4**	*O. tylus****	0.463	0.478	0.168	0.000 – 0.008			
**5**	*O. washingtoniae*****	0.478	0.497	0.193	0.127	0.000		
**6**	*O. dactyloni* (SVN: 3, Saudi Arabia)	0.481	0.488	0.189	0.186	0.180	0.000	
**7**	*O. pratensis* (SVN: 40, USA)	0.472	0.475	0.152	0.112	0.137	0.186	0.000

* It represents the average genetic distance value of 16 samples of *O. coniferarum* (sample voucher no./SVN: 9, 18, 22, 45, 57, 58, 68, 79, 81, 93, 116, 118, 119, 127, 128, and 150; Table S1), showing the same haplotype from Saudi Arabia.

** It represents the average range of genetic distance value of 29 samples of *O. afrasiaticus* ((SVN: 4, 7, 33, 34, 39, 46, 49, 50, 66, 82, 85, 86, 89, 91, 94, 99, 102, 106, 109, 110, 112, 129, 157, 167, 170, 171, 173, and 176; Table S1 & accession no: DQ656437), showing the same haplotype from Israel, Pakistan, Saudi Arabia, and Yemen. The SVN: 39 was previously claimed as *O. pratensis* from Saudi Arabia [1].

*** It represents the average range of genetic distance value of nine samples of *O. tylus* ((SVN: 16, 51, 54, 61, 67, 98, 125, and 143; Table S1; accession no: MK640235), showing the same haplotype from India, Pakistan, and Saudi Arabia.

**** It represents the average genetic distance value of 10 samples of *O. washingtoniae* (sample voucher no./SVN: 2, 83, 84, 87, 88, 95, 124, 133, 168, and 175; Table S1), showing the same haplotype from Saudi Arabia.

Table S3. Genetic divergence (pairwise p-distance) based on nine COI partial sequences, either obtained in the present study or retrieved from GenBank, among six *Oligonychus* species (Acari: Prostigmata: Tetranychidae), collected/analyzed from Saudi Arabia and the USA.

**No.**	***Oligonychus* species**	**1**	**2**	**3**	**4**	**5**	**6**
**1**	*O. punicae**	0.000					
**2**	*O. afrasiaticus***	0.165	0.000 – 0.029				
**3**	*O. tylus****	0.162	0.142	0.000 – 0.003			
**4**	*O. washingtoniae* (SVN: 2, Saudi Arabia)	0.147	0.106	0.127	0.000		
**5**	*O. pratensis* (SVN: 40, USA)	0.142	0.094	0.124	0.088	0.000	
**6**	*O. dactyloni*****	0.136	0.130	0.127	0.121	0.118	0.000

* It represents the average genetic distance value of three samples of *O. punicae* (sample voucher no./SVN: 144 and 145; Table S1; accession no: MZ425483), showing the same haplotype from Saudi Arabia.

** It represents the average range of genetic distance value of eight samples of *O. afrasiaticus* ((SVN: 49, 50, 91, 102, 109, 110, and 112; Table S1; accession no: DQ656482), showing the same haplotype from Israel, Saudi Arabia, and Yemen.

*** It represents the average range of genetic distance value of four samples of *O. tylus* ((SVN: 54, 61, and 143; Table S1; accession no: KU323485), showing the same haplotype from India and Saudi Arabia

**** It represents the average genetic distance value of two samples of *O. dactyloni* (sample voucher no./SVN: 3 and 11; Table S1), showing the same haplotype from Saudi Arabia.

Table S4. Genetic divergence (pairwise p-distance) based on 38 ITS2 partial sequences, either obtained in the present study or retrieved from GenBank, showing variations among various populations of *Oligonychus afrasiaticus* and *O. tylus* (Acari: Prostigmata: Tetranychidae), collected/analyzed from Israel, India, Pakistan, Saudi Arabia, and Yemen.

**No.**	***Oligonychus* species**	**1**	**2**	**3**	**4**	**5**	**6**	**7**	**8**	**9**	**10**	**11**	**12**	**13**
**1**	*O. afrasiaticus* **(H1)***	**0.000**												
**2**	*O. afrasiaticus* **(H2)****	0.006	0.000											
**3**	*O. afrasiaticus* **(H3)*****	0.004	0.006	0.000										
**4**	*O. afrasiaticus* (SVN: 34, Saudi Arabia) **(H4)**	0.006	0.004	0.004	0.000									
**5**	*O. afrasiaticus* (SVN: 171, Saudi Arabia) **(H5)**	0.006	0.004	0.006	0.000	0.000								
**6**	*O. afrasiaticus* **(H6)******	0.008	0.002	0.008	0.002	0.002	0.000							
**7**	*O. afrasiaticus* (SVN: 66, Pakistan) **(H7)**	0.006	0.008	0.010	0.008	0.008	0.010	0.000						
**8**	*O. afrasiaticus* (SVN: 110, Saudi Arabia) **(H8)**	0.006	0.004	0.010	0.004	0.004	0.002	0.008	0.000					
**9**	*O. afrasiaticus* (SVN: 173, Saudi Arabia) **(H9)**	0.010	0.008	0.004	0.006	0.008	0.010	0.012	**0.012**	0.000				
**10**	*O. tylus* (MK640235, India) **(H1)**	0.188	0.188	0.189	0.164	0.192	0.190	0.190	0.190	0.195	**0.000**			
**11**	*O. tylus* **(H2)*******	0.188	0.188	0.189	0.164	0.192	0.190	0.190	0.190	0.195	0.004	0.000		
**12**	*O. tylus* (SVN: 143, Saudi Arabia) **(H3)**	0.190	0.190	0.191	0.166	0.194	0.192	0.192	0.192	0.197	**0.008**	0.004	0.000	
**13**	*O. tylus* **(H4)********	0.188	0.188	0.189	0.164	0.192	0.190	0.190	0.190	0.195	0.004	0.000	0.004	0.000

* It represents the ITS2 haplotype 1 of *O. afrasiaticus* that contains 10 samples (sample voucher no./SVN: 4, 7, 39, 82, 85, 86, 89, 106, and 170; Table S1; accession no: DQ 656437), collected from Israel and Saudi Arabia.
** It represents the ITS2 haplotype 2 of *O. afrasiaticus* that contains eight samples (SVN: 33, 46, 49, 50, 91, 94, 102, and 176; Table S1), collected from Saudi Arabia and Yemen.
*** It represents the ITS2 haplotype 3 of *O. afrasiaticus* that contains three samples (SVN: 99, 157, and 167; Table S1), collected from Saudi Arabia.
**** It represents the ITS2 haplotype 6 of *O. afrasiaticus* that contains three samples (SVN: 109, 112, and 129; Table S1), collected from Saudi Arabia.
***** It represents the ITS2 haplotype 2 of *O. tylus* that contains five samples (SVN: 51, 54, 61, 67, and 98; Table S1), collected from Pakistan and Saudi Arabia.
****** It represents the ITS2 haplotype 4 of *O. tylus* that contains two samples (SVN: 16 and 125; Table S1), collected from Saudi Arabia.
- The yellow highlighted section represents nine different haplotypes of *O. afrasiaticus*.
- The blue highlighted section represents four different haplotypes of *O. tylus*.

Table S5. Genetic divergence (pairwise p-distance) based on 12 COI partial sequences, either obtained in the present study or retrieved from GenBank, showing variations among various populations of *Oligonychus afrasiaticus* and *O. tylus* (Acari: Prostigmata: Tetranychidae), collected/analyzed from Israel, India, Saudi Arabia, and Yemen.

**No.**	***Oligonychus* species**	**1**	**2**	**3**	**4**	**5**
**1**	*O. afrasiaticus* (DQ656482, Israel) **(H1)**	0.000				
**2**	*O. afrasiaticus* **(H2)***	0.029	0.000			
**3**	*O. afrasiaticus* **(H3)****	0.020	0.010	0.000		
**4**	*O. tylus* **(H1)*****	0.124	0.135	0.132	0.000	
**5**	*O. tylus* **(H2)******	0.124	0.141	0.139	0.003	0.000

* It represents the COI haplotype 2 of *O. afrasiaticus* that contains five samples (sample voucher no./SVN: 91, 102, 109, 110, and 112; Table S1), collected from Saudi Arabia.

** It represents the COI haplotype 3 of *O. afrasiaticus* that contains two samples (SVN: 49 and 50; Table S1), collected from Yemen.

*** It represents the COI haplotype 1 of *O. tylus* that contains two samples (SVN: 54; Table S1; accession no: KU323485), collected from India and Saudi Arabia.

**** It represents the COI haplotype 2 of *O. tylus* that contains two samples (SVN: 61 and 143; Table S1), collected from Saudi Arabia.

- The yellow highlighted section represents three different haplotypes of *O. afrasiaticus*.
- The blue highlighted section represents two different haplotypes of *O. tylus*.

Table S6. Genetic divergence (pairwise p-distance) based on ITS2 sequences among 15 *Oligonychus* species (Acari: Prostigmata: Tetranychidae), either obtained in the present study or retrieved from GenBank, analyzed from India, Mexico, Saudi Arabia, and the USA.

No.	***Oligonychus* species**	1	2	3	4	5	6	7	8	9	10	11	12	13	14	15
**1**	*O. punicae* (MZ435900, Saudi Arabia)	0.000														
**2**	*O. coniferarum* (SVN: 22, Saudi Arabia)	0.067	0.000													
**3**	*Oligonychus* sp. (KC352302, Mexico)*	0.104	0.081	0.000												
**4**	*O. perseae* (SVN. 53, Mexico)	0.296	0.289	0.326	0.000											
**5**	*O. afrasiaticus* (SVN: 91, Saudi Arabia)	0.470	0.481	0.493	0.500	0.000										
**6**	*O. tylus* (SVN. 54, Saudi Arabia)	0.452	0.459	0.481	0.489	0.115	0.000									
**7**	*O. washingtoniae* (SVN: 2, Saudi Arabia)	0.448	0.456	0.478	0.489	0.133	0.078	0.000								
**8**	*O. dactyloni* (SVN: 3, Saudi Arabia)	0.467	0.478	0.493	0.511	0.181	0.144	0.144	0.000							
**9**	*O. pratensis* (SVN: 40, USA)	0.470	0.478	0.496	0.485	0.141	0.089	0.115	0.148	0.000						
**10**	*O. indicus* (MN190324, India)	0.470	0.478	0.489	0.493	0.122	0.063	0.081	0.115	0.074	0.000					
**11**	*Oligonychus* sp. (MK386956, India)	0.470	0.463	0.481	0.507	0.152	0.096	0.133	0.141	0.126	0.081	0.000				
**12**	*Oligonychus* sp. (OL830813, India)	0.467	0.456	0.474	0.496	0.144	0.081	0.111	0.130	0.104	0.067	0.044	0.000			
**13**	*Oligonychus* sp. (MG677944, India)	0.456	0.463	0.485	0.493	0.115	0.056	0.070	0.141	0.078	0.059	0.104	0.081	0.000		
**14**	*Oligonychus* sp. (MG429138, India)	0.448	0.463	0.478	0.481	0.115	0.044	0.093	0.137	0.100	0.078	0.104	0.089	0.074	0.000	
**15**	*O. oryzae* (OP214345, India)	0.470	0.463	0.481	0.507	0.152	0.096	0.126	0.137	0.119	0.081	0.037	0.037	0.104	0.104	0.000

* It represents a cryptic *Oligonychus* sp., previously claimed as *O. punicae* in Mexico [2].

Table S7. Genetic divergence (pairwise p-distance) based on COI sequences among 32 *Oligonychus* species (Acari: Prostigmata: Tetranychidae), either obtained in the present study or retrieved from GenBank.

No.	***Oligonychus* species**	1	2	3	4	5	6	7	8	9	10	11	12	13	14	15	16	17	18	19	20	21	22	23	24	25	26	27	28	29	30	31	32
**1**	*O. punicae* (MZ425483, Saudi Arabia)	0.000																															
**2**	*Oligonychus* sp. (SVN: 52, Mexico)*	0.104	0.000																														
**3**	*O. ununguis* (AB683664, Japan)	0.107	0.078	0.000																													
**4**	*O. ununguis* (GU329963, China)	0.087	0.104	0.110	0.000																												
**5**	*O. ununguis* (X80865, France)	0.097	0.091	0.065	0.068	0.000																											
**6**	*O. gotohi* (AB683669, Japan)	0.074	0.074	0.084	0.087	0.087	0.000																										
**7**	*O. castaneae* (AB683666, Japan)	0.084	0.084	0.061	0.074	0.055	0.074	0.000																									
**8**	*O. pustulosus* (AB683655, Japan)	0.091	0.087	0.084	0.068	0.071	0.074	0.081	0.000																								
**9**	*O. tsudomei* (AB683659, Japan)	0.091	0.087	0.097	0.084	0.091	0.087	0.084	0.084	0.000																							
**10**	*O. ilicis* (AB683660, Japan)	0.094	0.087	0.084	0.087	0.078	0.078	0.087	0.081	0.094	0.000																						
**11**	*O. amiensis* (AB683673, Japan)	0.107	0.100	0.091	0.094	0.091	0.091	0.081	0.081	0.087	0.078	0.000																					
**12**	*O. hondoensis* (AB683658, Japan)	0.110	0.107	0.091	0.107	0.078	0.113	0.078	0.081	0.097	0.091	0.097	0.000																				
**13**	*O. perditus* (AB683665, Japan)	0.120	0.081	0.071	0.113	0.087	0.074	0.084	0.094	0.091	0.097	0.107	0.113	0.000																			
**14**	*O. clavatus* (AB683653, Japan)	0.084	0.100	0.097	0.065	0.074	0.074	0.068	0.081	0.081	0.068	0.087	0.087	0.097	0.000																		
**15**	*O. neocastaneae* (LC341206, Japan)	0.087	0.100	0.104	0.032	0.081	0.084	0.065	0.091	0.087	0.091	0.110	0.110	0.113	0.071	0.000																	
**16**	*O. karamatus* (AB683656, Japan)	0.097	0.081	0.091	0.074	0.074	0.081	0.068	0.078	0.084	0.068	0.081	0.087	0.084	0.071	0.071	0.000																
**17**	*O. camelliae* (AB683662, Japan)	0.113	0.078	0.087	0.097	0.084	0.084	0.084	0.078	0.087	0.078	0.094	0.094	0.087	0.087	0.107	0.087	0.000															
**18**	*O. platani* (X80866, France)	0.100	0.100	0.100	0.081	0.071	0.094	0.081	0.071	0.084	0.068	0.081	0.094	0.107	0.074	0.091	0.074	0.084	0.000														
**19**	*Oligonychus* sp. (MF462136, Australia)	0.094	0.074	0.071	0.087	0.078	0.074	0.065	0.068	0.087	0.078	0.091	0.091	0.081	0.091	0.087	0.074	0.081	0.091	0.000													
**20**	*O. perseae* (DQ656485, Israel)	0.129	0.117	0.097	0.142	0.107	0.117	0.107	0.107	0.133	0.117	0.123	0.129	0.123	0.133	0.146	0.123	0.123	0.136	0.107	0.000												
**21**	*O. biharensis* (OP363224, India)	0.149	0.149	0.159	0.123	0.120	0.149	0.133	0.133	0.139	0.139	0.155	0.142	0.159	0.123	0.133	0.120	0.155	0.123	0.139	0.159	0.000											
**22**	*O. indicus* (KC009700, India)	0.162	0.159	0.152	0.155	0.152	0.159	0.146	0.152	0.146	0.159	0.165	0.159	0.175	0.146	0.155	0.172	0.162	0.162	0.152	0.155	0.136	0.000										
**23**	*O. orthius* (AB683675, Japan)	0.149	0.126	0.117	0.129	0.123	0.117	0.126	0.091	0.123	0.113	0.129	0.117	0.139	0.126	0.136	0.123	0.120	0.117	0.113	0.142	0.123	0.104	0.000									
**24**	*O. rubicundus* (AB683681, Japan)	0.149	0.133	0.129	0.139	0.123	0.126	0.133	0.110	0.133	0.123	0.139	0.136	0.133	0.133	0.152	0.123	0.126	0.133	0.110	0.120	0.113	0.107	0.068	0.000								
**25**	*O. modestus* (AB683677, Japan)	0.149	0.139	0.146	0.152	0.149	0.126	0.139	0.120	0.133	0.139	0.129	0.146	0.149	0.142	0.159	0.133	0.136	0.142	0.107	0.165	0.142	0.113	0.097	0.100	0.000							
**26**	*O. grypus* (MW491838, India)	0.139	0.159	0.142	0.152	0.146	0.155	0.136	0.136	0.149	0.129	0.149	0.133	0.172	0.133	0.146	0.136	0.155	0.133	0.117	0.162	0.113	0.110	0.091	0.097	0.117	0.000						
**27**	*Oligonychus* sp. (KP180428, USA)	0.146	0.142	0.146	0.155	0.136	0.139	0.139	0.142	0.155	0.142	0.162	0.152	0.162	0.139	0.162	0.142	0.152	0.139	0.126	0.149	0.107	0.136	0.113	0.091	0.126	0.123	0.000					
**28**	*O. afrasiaticus* (SVN: 91, Saudi Arabia)	0.165	0.146	0.149	0.168	0.155	0.155	0.146	0.149	0.146	0.149	0.149	0.146	0.159	0.155	0.168	0.146	0.146	0.136	0.142	0.155	0.123	0.123	0.100	0.094	0.117	0.136	0.133	0.000				
**29**	*O. tylus* (SVN: 54, Saudi Arabia)	0.165	0.159	0.155	0.152	0.139	0.162	0.165	0.139	0.155	0.165	0.181	0.159	0.146	0.142	0.162	0.172	0.162	0.159	0.168	0.172	0.139	0.117	0.110	0.120	0.136	0.142	0.139	0.142	0.000			
**30**	*O. washingtoniae* (SVN: 2, Saudi Arabia)	0.152	0.129	0.142	0.155	0.152	0.139	0.146	0.129	0.146	0.136	0.162	0.146	0.159	0.152	0.149	0.146	0.152	0.146	0.126	0.152	0.126	0.104	0.084	0.074	0.107	0.100	0.091	0.104	0.133	0.000		
**31**	*O. pratensis* (SVN: 40, USA)	0.142	0.159	0.139	0.162	0.142	0.159	0.142	0.136	0.162	0.136	0.172	0.152	0.162	0.142	0.159	0.146	0.162	0.142	0.139	0.152	0.142	0.120	0.100	0.094	0.133	0.117	0.113	0.097	0.129	0.087	0.000	
**32**	*O. dactyloni* (SVN: 3, Saudi Arabia)	0.133	0.139	0.149	0.139	0.136	0.139	0.133	0.133	0.129	0.136	0.165	0.155	0.159	0.129	0.126	0.133	0.152	0.129	0.136	0.152	0.126	0.123	0.117	0.113	0.126	0.107	0.123	0.136	0.129	0.120	0.120	0.000

* It represents a cryptic *Oligonychus* sp., previously claimed as *O. punicae* in Mexico [2].

- The yellow highlighted section represents three GenBank sequences of the claimed *O. ununguis* from Japan, China, and France that need to be revisited to confirm the taxonomic identity of their voucher specimens.

References

1. Alatawi, F.J.; Kamran, M. Spider mites (Acari: Tetranychidae) of Saudi Arabia: two new species, new records and a key to all known species. *Journal of Natural History* **2018**, *52*, 429-455, doi:10.1080/00222933.2018.1434251.
2. Mushtaq, H.M.S.; Kamran, M.; Saleh, A.A.; Alatawi, F.J. Evidence for Reconsidering the Taxonomic Status of Closely Related *Oligonychus* Species in punicae Complex (Acari: Prostigmata: Tetranychidae). *Insects* **2023**, *14*, 1-16, doi:10.3390/insects14010003.
